# 4-[3-(2*H*-Benzotriazol-2-yl)prop­oxy]-3-methoxy­benzaldehyde

**DOI:** 10.1107/S1600536810015461

**Published:** 2010-05-08

**Authors:** Lei Jin, Cheng-He Zhou

**Affiliations:** a Laboratory of Bioorganic & Medicinal Chemistry, School of Chemistry & Chemical Engineering, Southwest University, Chongqing 400715, People’s Republic of China; b and School of Pharmaceutical Sciences, Southwest University, Chongqing 400715, People’s Republic of China

## Abstract

In the title compound, C_17_H_17_N_3_O_3_, the 3-methoxy­benzalde­hyde group and the benzotriazole fragment are connected through a flexible oxypropyl chain. The O—C—C—C torsion angle in the central link is −63.9 (2)°, while the plane of the benzene ring of the 3-methoxy­benzaldehyde substituent forms a dihedral angle of 56.4 (4)° with the benzotriazole plane.

## Related literature

For general background to the biological activity of 1*H*-benzotriazole and its derivatives, see: Al-Soud *et al.* (2003 ▶); Khalafi-Nezhad *et al.* (2005 ▶); Nanjunda Swamy *et al.* (2006 ▶). For a related structure, see: Jin *et al.* (2009 ▶). 
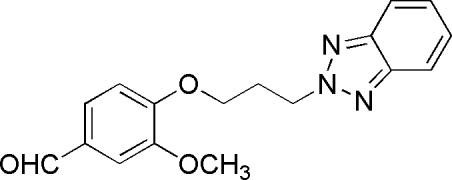


## Experimental

### 

#### Crystal data

C_17_H_17_N_3_O_3_
*M* *_r_* = 311.34
Monoclinic, 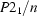

*a* = 11.328 (2) Å
*b* = 8.1278 (16) Å
*c* = 16.156 (3) Å
β = 100.301 (3)°
*V* = 1463.6 (5) Å^3^
*Z* = 4
Mo *K*α radiation
μ = 0.10 mm^−1^
*T* = 173 K
0.34 × 0.20 × 0.18 mm

#### Data collection

Bruker SMART diffractometer
Absorption correction: multi-scan (*SADABS*; Sheldrick, 1996 ▶) *T*_min_ = 0.967, *T*_max_ = 0.982
7400 measured reflections
2716 independent reflections
2257 reflections with *I* > 2σ(*I*)
*R*_int_ = 0.030

#### Refinement

*R*[*F* ^2^ > 2σ(*F* ^2^)] = 0.043
*wR*(*F* ^2^) = 0.111
*S* = 1.02
2716 reflections
209 parameters
H-atom parameters constrained
Δρ_max_ = 0.20 e Å^−3^
Δρ_min_ = −0.23 e Å^−3^

### 

Data collection: *SMART* (Bruker, 2001 ▶); cell refinement: *SAINT-Plus* (Bruker, 2001 ▶); data reduction: *SAINT-Plus*; program(s) used to solve structure: *SHELXS97* (Sheldrick, 2008 ▶); program(s) used to refine structure: *SHELXL97* (Sheldrick, 2008 ▶); molecular graphics: *SHELXTL* (Sheldrick, 2008 ▶); software used to prepare material for publication: *SHELXTL* and *publCIF* (Westrip, 2010 ▶).

## Supplementary Material

Crystal structure: contains datablocks I, global. DOI: 10.1107/S1600536810015461/ya2115sup1.cif

Structure factors: contains datablocks I. DOI: 10.1107/S1600536810015461/ya2115Isup2.hkl

Additional supplementary materials:  crystallographic information; 3D view; checkCIF report

## Figures and Tables

**Fig. 1.**
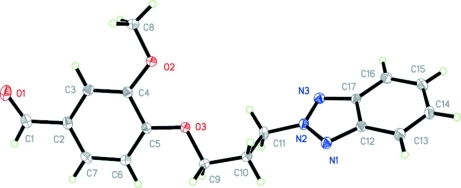
Molecular structure of the title compound, showing atom-numbering scheme. Displacement ellipsoids are drawn at the 30% probability level.

## supplementary crystallographic information

### Comment

The incorporation of azole nucleus is an important synthetic strategy in drug discovery. The high therapeutic properties of the related drugs have encouraged the medicinal chemists to synthesize large number of novel chemotherapeutic agents. 1*H*-Benzotriazole and many of its derivatives exhibit important biological properties, some are showing anti-inflammatory, antiviral, antifungal, antineoplastic and antidepressant activities (Al-Soud *et al.*, 2003; Nanjunda Swamy *et al.*, 2006). Recently, the structure of aralkyl nitroimidazole ether, which shows inhibitory effects on several types of pathogenic bacteria, has been published (Khalafi-Nezhad *et al.*, 2005; Jin *et al., *2009). Taking into account promising therapeutic applications of benzotriazole derivatives, we are focusing on the development of new drugs belonging to this class. Herein we report the crystal structure of the title compound (Fig. 1).

The 3-methoxybenzaldehyde group and benzotriazole fragment in the molecule of the title compound are connected through the flexible oxypropyl chain. The O3—C9—C10—C11 torsion angle in the central link is equal to -63.9 (2)°, whereas the planes of the benzene ring C2—C7 and benzotriazole system N1—N3, C12—C17 form the dihedral angle of 56.4 (4)°.

### Experimental

A solution of benzotriazole (0.119 g, 1 mmol), 4-(3-bromopropoxy)-3-methoxy benzaldehyde (0.273 g, 1 mmol) and triethyl amine (1.01 g, 0.01 mol) in anhydrous MeCN (40 ml) was refluxed for approximately 10 h, when TLC monitoring indicated disappearance of benzotriazole; the solvent was then evaporated and the crude mixture was suspended in 200 ml of water. The organic materials were extracted with CH_2_Cl_2_ (2 × 150 ml). Both portions were combined, dried over anhydrous Na_2_SO_4_, and then evaporated to give the crude product, further purified by column chromatography on silica gel with EtOAc to afford the title compound (yield: 0.241 g, 78%; colourless solid; Mp. 411–413 K). Single crystal used in X-ray diffraction analysis was obtained at room temperature by slow evaporation of the solution of title compound in the mixture of ethyl acetate and dichloromethane.

### Refinement

Hydrogen atoms were placed in geometrically calculated positions (C—H 0.95 Å for aromatic and formyl, 0.99 Å for methylene and 0.98 Å for methyl) and included in the refinement in a riding motion approximation with U*iso*(H) = 1.2U*eq*(C) [for methyl groups U*iso*(H) = 1.5U*eq*(C)].

### Crystal data

**Table:**

C_17_H_17_N_3_O_3_	*F*(000) = 656
*M**_r_* = 311.34	*D*_x_ = 1.413 Mg m^−^^3^
Monoclinic, *P*2_1_/*n*	Mo *K*α radiation, λ = 0.71073 Å
Hall symbol: -P 2yn	Cell parameters from 2314 reflections
*a* = 11.328 (2) Å	θ = 2.4–26.8°
*b* = 8.1278 (16) Å	µ = 0.10 mm^−^^1^
*c* = 16.156 (3) Å	*T* = 173 K
β = 100.301 (3)°	Block, colourless
*V* = 1463.6 (5) Å^3^	0.34 × 0.20 × 0.18 mm
*Z* = 4	

### Data collection

**Table:**

Bruker SMART diffractometer	2716 independent reflections
Radiation source: fine-focus sealed tube	2257 reflections with *I* > 2σ(*I*)
graphite	*R*_int_ = 0.030
phi and ω scans	θ_max_ = 25.5°, θ_min_ = 2.0°
Absorption correction: multi-scan (*SADABS*; Sheldrick, 1996)	*h* = −13→11
*T*_min_ = 0.967, *T*_max_ = 0.982	*k* = −9→9
7400 measured reflections	*l* = −14→19

### Refinement

**Table:**

Refinement on *F*^2^	Primary atom site location: structure-invariant direct methods
Least-squares matrix: full	Secondary atom site location: difference Fourier map
*R*[*F*^2^ > 2σ(*F*^2^)] = 0.043	Hydrogen site location: inferred from neighbouring sites
*wR*(*F*^2^) = 0.111	H-atom parameters constrained
*S* = 1.02	*w* = 1/[σ^2^(*F*_o_^2^) + (0.0598*P*)^2^ + 0.2971*P*] where *P* = (*F*_o_^2^ + 2*F*_c_^2^)/3
2716 reflections	(Δ/σ)_max_ = 0.002
209 parameters	Δρ_max_ = 0.20 e Å^−^^3^
0 restraints	Δρ_min_ = −0.23 e Å^−^^3^

### Special details

**Table:**

Geometry. All esds (except the esd in the dihedral angle between two l.s. planes) are estimated using the full covariance matrix. The cell esds are taken into account individually in the estimation of esds in distances, angles and torsion angles; correlations between esds in cell parameters are only used when they are defined by crystal symmetry. An approximate (isotropic) treatment of cell esds is used for estimating esds involving l.s. planes.
Refinement. Refinement of *F*^2^ against ALL reflections. The weighted *R*-factor wR and goodness of fit *S* are based on *F*^2^, conventional *R*-factors *R* are based on *F*, with *F* set to zero for negative *F*^2^. The threshold expression of *F*^2^ > σ(*F*^2^) is used only for calculating *R*-factors(gt) etc. and is not relevant to the choice of reflections for refinement. *R*-factors based on *F*^2^ are statistically about twice as large as those based on *F*, and *R*- factors based on ALL data will be even larger.

### Fractional atomic coordinates and isotropic or equivalent isotropic displacement parameters (Å^2^)

**Table:**

	*x*	*y*	*z*	*U*_iso_*/*U*_eq_	
C1	0.30868 (15)	0.3509 (2)	−0.18483 (10)	0.0304 (4)	
H1	0.2604	0.4172	−0.2261	0.036*	
C2	0.32240 (13)	0.40484 (19)	−0.09726 (9)	0.0252 (4)	
C3	0.38488 (13)	0.30819 (19)	−0.03210 (9)	0.0247 (4)	
H3	0.4194	0.2068	−0.0447	0.030*	
C4	0.39637 (13)	0.35957 (18)	0.04991 (9)	0.0233 (3)	
C5	0.34662 (13)	0.51165 (19)	0.06848 (9)	0.0231 (3)	
C6	0.28450 (14)	0.60657 (19)	0.00395 (10)	0.0266 (4)	
H6	0.2503	0.7085	0.0161	0.032*	
C7	0.27228 (14)	0.5522 (2)	−0.07870 (10)	0.0283 (4)	
H7	0.2290	0.6171	−0.1229	0.034*	
C8	0.50723 (16)	0.12187 (19)	0.10204 (11)	0.0336 (4)	
H8A	0.4451	0.0477	0.0730	0.050*	
H8B	0.5457	0.0717	0.1552	0.050*	
H8C	0.5675	0.1412	0.0665	0.050*	
C9	0.33302 (15)	0.71678 (19)	0.17160 (10)	0.0269 (4)	
H9A	0.2451	0.7318	0.1563	0.032*	
H9B	0.3725	0.7982	0.1400	0.032*	
C10	0.37338 (14)	0.74091 (19)	0.26499 (10)	0.0260 (4)	
H10A	0.3584	0.8563	0.2798	0.031*	
H10B	0.4607	0.7203	0.2798	0.031*	
C11	0.30818 (14)	0.6269 (2)	0.31527 (9)	0.0283 (4)	
H11A	0.2212	0.6512	0.3022	0.034*	
H11B	0.3198	0.5119	0.2981	0.034*	
C12	0.42907 (13)	0.73940 (19)	0.52211 (10)	0.0236 (4)	
C13	0.48871 (14)	0.8333 (2)	0.59075 (10)	0.0288 (4)	
H13	0.5255	0.9357	0.5829	0.035*	
C14	0.49051 (14)	0.7689 (2)	0.66857 (10)	0.0296 (4)	
H14	0.5296	0.8285	0.7162	0.036*	
C15	0.43636 (14)	0.6163 (2)	0.68129 (10)	0.0305 (4)	
H15	0.4408	0.5765	0.7371	0.037*	
C16	0.37797 (15)	0.5246 (2)	0.61590 (10)	0.0297 (4)	
H16	0.3416	0.4224	0.6248	0.036*	
C17	0.37411 (13)	0.58889 (18)	0.53437 (10)	0.0230 (3)	
N1	0.41205 (12)	0.77182 (16)	0.43877 (8)	0.0270 (3)	
N2	0.34983 (11)	0.64249 (15)	0.40576 (8)	0.0238 (3)	
N3	0.32342 (11)	0.52833 (16)	0.45819 (8)	0.0268 (3)	
O1	0.35353 (11)	0.22907 (16)	−0.20936 (7)	0.0390 (3)	
O2	0.45409 (10)	0.27407 (13)	0.11862 (7)	0.0294 (3)	
O3	0.36540 (10)	0.55293 (13)	0.15124 (6)	0.0276 (3)	

### Atomic displacement parameters (Å^2^)

**Table:**

	*U*^11^	*U*^22^	*U*^33^	*U*^12^	*U*^13^	*U*^23^
C1	0.0309 (9)	0.0366 (10)	0.0234 (9)	−0.0077 (7)	0.0045 (7)	0.0009 (7)
C2	0.0253 (8)	0.0294 (9)	0.0211 (8)	−0.0072 (7)	0.0044 (6)	−0.0002 (7)
C3	0.0268 (8)	0.0220 (8)	0.0261 (9)	−0.0023 (6)	0.0065 (7)	−0.0018 (7)
C4	0.0248 (8)	0.0234 (8)	0.0216 (8)	−0.0019 (6)	0.0036 (6)	0.0017 (6)
C5	0.0251 (8)	0.0246 (8)	0.0197 (8)	−0.0027 (6)	0.0043 (6)	0.0001 (6)
C6	0.0308 (9)	0.0238 (8)	0.0252 (9)	0.0024 (7)	0.0048 (7)	0.0007 (7)
C7	0.0306 (9)	0.0300 (9)	0.0235 (9)	−0.0018 (7)	0.0022 (7)	0.0062 (7)
C8	0.0442 (10)	0.0256 (9)	0.0302 (9)	0.0090 (7)	0.0047 (8)	0.0024 (7)
C9	0.0361 (9)	0.0211 (8)	0.0235 (9)	0.0044 (7)	0.0053 (7)	−0.0003 (6)
C10	0.0326 (9)	0.0200 (8)	0.0248 (9)	0.0015 (6)	0.0035 (7)	−0.0016 (6)
C11	0.0282 (8)	0.0353 (9)	0.0204 (8)	−0.0035 (7)	0.0014 (6)	−0.0044 (7)
C12	0.0228 (8)	0.0257 (8)	0.0221 (8)	0.0018 (6)	0.0031 (6)	−0.0005 (6)
C13	0.0291 (9)	0.0275 (8)	0.0284 (9)	−0.0042 (7)	0.0014 (7)	−0.0023 (7)
C14	0.0283 (9)	0.0362 (10)	0.0226 (9)	0.0002 (7)	−0.0001 (7)	−0.0048 (7)
C15	0.0311 (9)	0.0368 (10)	0.0229 (9)	0.0029 (7)	0.0034 (7)	0.0043 (7)
C16	0.0332 (9)	0.0271 (9)	0.0294 (9)	−0.0010 (7)	0.0067 (7)	0.0038 (7)
C17	0.0216 (8)	0.0225 (8)	0.0244 (8)	0.0032 (6)	0.0026 (6)	−0.0021 (6)
N1	0.0311 (8)	0.0249 (7)	0.0238 (8)	−0.0048 (6)	0.0023 (6)	−0.0032 (6)
N2	0.0262 (7)	0.0238 (7)	0.0214 (7)	−0.0021 (5)	0.0041 (5)	−0.0019 (5)
N3	0.0293 (7)	0.0246 (7)	0.0265 (8)	−0.0008 (5)	0.0053 (6)	−0.0006 (6)
O1	0.0457 (8)	0.0429 (8)	0.0302 (7)	−0.0049 (6)	0.0113 (6)	−0.0098 (6)
O2	0.0402 (7)	0.0242 (6)	0.0225 (6)	0.0084 (5)	0.0022 (5)	0.0009 (5)
O3	0.0394 (7)	0.0226 (6)	0.0199 (6)	0.0061 (5)	0.0029 (5)	−0.0016 (4)

### Geometric parameters (Å, °)

**Table:**

C1—O1	1.211 (2)	C9—H9B	0.9900
C1—C2	1.463 (2)	C10—C11	1.510 (2)
C1—H1	0.9500	C10—H10A	0.9900
C2—C7	1.381 (2)	C10—H10B	0.9900
C2—C3	1.400 (2)	C11—N2	1.459 (2)
C3—C4	1.373 (2)	C11—H11A	0.9900
C3—H3	0.9500	C11—H11B	0.9900
C4—O2	1.3723 (18)	C12—N1	1.352 (2)
C4—C5	1.413 (2)	C12—C17	1.403 (2)
C5—O3	1.3580 (18)	C12—C13	1.414 (2)
C5—C6	1.384 (2)	C13—C14	1.359 (2)
C6—C7	1.390 (2)	C13—H13	0.9500
C6—H6	0.9500	C14—C15	1.415 (2)
C7—H7	0.9500	C14—H14	0.9500
C8—O2	1.4220 (18)	C15—C16	1.363 (2)
C8—H8A	0.9800	C15—H15	0.9500
C8—H8B	0.9800	C16—C17	1.410 (2)
C8—H8C	0.9800	C16—H16	0.9500
C9—O3	1.4350 (18)	C17—N3	1.3543 (19)
C9—C10	1.509 (2)	N1—N2	1.3235 (17)
C9—H9A	0.9900	N2—N3	1.3261 (18)
			
O1—C1—C2	125.67 (16)	C11—C10—H10A	109.3
O1—C1—H1	117.2	C9—C10—H10B	109.3
C2—C1—H1	117.2	C11—C10—H10B	109.3
C7—C2—C3	119.71 (14)	H10A—C10—H10B	108.0
C7—C2—C1	119.55 (15)	N2—C11—C10	112.58 (13)
C3—C2—C1	120.74 (15)	N2—C11—H11A	109.1
C4—C3—C2	120.20 (15)	C10—C11—H11A	109.1
C4—C3—H3	119.9	N2—C11—H11B	109.1
C2—C3—H3	119.9	C10—C11—H11B	109.1
O2—C4—C3	125.16 (14)	H11A—C11—H11B	107.8
O2—C4—C5	114.95 (13)	N1—C12—C17	108.84 (13)
C3—C4—C5	119.89 (14)	N1—C12—C13	129.72 (15)
O3—C5—C6	124.97 (14)	C17—C12—C13	121.44 (15)
O3—C5—C4	115.28 (13)	C14—C13—C12	116.34 (15)
C6—C5—C4	119.76 (14)	C14—C13—H13	121.8
C5—C6—C7	119.77 (15)	C12—C13—H13	121.8
C5—C6—H6	120.1	C13—C14—C15	122.49 (15)
C7—C6—H6	120.1	C13—C14—H14	118.8
C2—C7—C6	120.67 (15)	C15—C14—H14	118.8
C2—C7—H7	119.7	C16—C15—C14	121.95 (15)
C6—C7—H7	119.7	C16—C15—H15	119.0
O2—C8—H8A	109.5	C14—C15—H15	119.0
O2—C8—H8B	109.5	C15—C16—C17	116.82 (15)
H8A—C8—H8B	109.5	C15—C16—H16	121.6
O2—C8—H8C	109.5	C17—C16—H16	121.6
H8A—C8—H8C	109.5	N3—C17—C12	108.39 (13)
H8B—C8—H8C	109.5	N3—C17—C16	130.65 (15)
O3—C9—C10	107.80 (12)	C12—C17—C16	120.96 (14)
O3—C9—H9A	110.1	N2—N1—C12	102.55 (12)
C10—C9—H9A	110.1	N1—N2—N3	117.59 (12)
O3—C9—H9B	110.1	N1—N2—C11	121.76 (12)
C10—C9—H9B	110.1	N3—N2—C11	120.63 (12)
H9A—C9—H9B	108.5	N2—N3—C17	102.64 (12)
C9—C10—C11	111.61 (13)	C4—O2—C8	116.40 (12)
C9—C10—H10A	109.3	C5—O3—C9	116.98 (11)
			
O1—C1—C2—C7	175.92 (16)	N1—C12—C17—N3	0.19 (17)
O1—C1—C2—C3	−4.6 (2)	C13—C12—C17—N3	179.28 (14)
C7—C2—C3—C4	0.0 (2)	N1—C12—C17—C16	179.98 (14)
C1—C2—C3—C4	−179.50 (14)	C13—C12—C17—C16	−0.9 (2)
C2—C3—C4—O2	179.09 (14)	C15—C16—C17—N3	−179.70 (15)
C2—C3—C4—C5	−1.0 (2)	C15—C16—C17—C12	0.5 (2)
O2—C4—C5—O3	1.43 (19)	C17—C12—N1—N2	−0.25 (16)
C3—C4—C5—O3	−178.48 (13)	C13—C12—N1—N2	−179.25 (16)
O2—C4—C5—C6	−178.82 (14)	C12—N1—N2—N3	0.26 (17)
C3—C4—C5—C6	1.3 (2)	C12—N1—N2—C11	178.47 (13)
O3—C5—C6—C7	179.23 (14)	C10—C11—N2—N1	17.3 (2)
C4—C5—C6—C7	−0.5 (2)	C10—C11—N2—N3	−164.51 (13)
C3—C2—C7—C6	0.8 (2)	N1—N2—N3—C17	−0.14 (17)
C1—C2—C7—C6	−179.71 (14)	C11—N2—N3—C17	−178.38 (13)
C5—C6—C7—C2	−0.5 (2)	C12—C17—N3—N2	−0.03 (16)
O3—C9—C10—C11	−63.91 (17)	C16—C17—N3—N2	−179.80 (15)
C9—C10—C11—N2	177.26 (13)	C3—C4—O2—C8	1.1 (2)
N1—C12—C13—C14	179.44 (15)	C5—C4—O2—C8	−178.83 (13)
C17—C12—C13—C14	0.6 (2)	C6—C5—O3—C9	−9.2 (2)
C12—C13—C14—C15	0.1 (2)	C4—C5—O3—C9	170.54 (13)
C13—C14—C15—C16	−0.5 (3)	C10—C9—O3—C5	−174.10 (12)
C14—C15—C16—C17	0.1 (2)		
